# Genetic testing of sperm donors at a human sperm bank in China

**DOI:** 10.3389/fendo.2022.942447

**Published:** 2022-09-20

**Authors:** Chuan Huang, Hui-Lan Wu, Wen-Jun Zhou, Zeng-Hui Huang, Xue-Feng Luo, Yu-Ling Tang, Qian Liu, Li-Qing Fan, Hong-Chuan Nie, Wen-Bing Zhu

**Affiliations:** ^1^ Clinical Research Center for Reproduction and Genetics in Hunan Province, Reproductive and Genetic Hospital of China International Trust and Investment Corporation (CITIC)-Xiangya, Changsha, China; ^2^ Institute of Reproductive and Stem Cell Engineering, Basic Medicine College, Central South University, Changsha, China

**Keywords:** Sperm bank, sperm donor, genetic testing, whole exome sequencing, specific gene test

## Abstract

**Background:**

In China, numerous human sperm banks only perform three-generation family history evaluation to exclude genetic diseases with clinical symptoms; therefore, many inherited risks cannot be detected before donor qualification even when a thorough genetic family history evaluation has been performed. Hence, the risk of recessive disease inheritance persists with the current eligibility guidelines in China regarding the donor selection process.

**Methods:**

Retrospective study that reviewed the genetic test analyses and clinical outcomes of young adult men who were qualified sperm donors at the Hunan Province Human Sperm Bank of China from January 1, 2018, to May 1, 2021. We included a total of 3231 qualified sperm donors: all donors underwent primary screening for thalassemia and glucose-6-phosphate dehydrogenase (G6PD) deficiency. Whereafter, 278 of donors underwent genetic testing for specific genes, and 43 donors underwent whole exome sequencing.

**Results:**

2.4% of 3231 qualified sperm donors might have thalassemia and 1.4% might have G6PD deficiency. Sperm donors with thalassemia and G6PD deficiency would be eliminated. Specific gene testing identified 7 of the 278 donors (2.5%) as carriers of at least one pathogenic or likely pathogenic variant in a gene, including 1.9% of 154 donors (3/154) as carrier variants in α-Like or β-Like globin genes, 17.6% of 17 donors (3/17) as carrier variants in *GJB2*, 12.5% of 8 donors (1/8) as carrier variants in *SMN1*. In addition, among the 43 sperm donors carrying the 111 pathogenic/likely pathogenic variants, eight (18.6%) were carriers of pathogenic variants of the *GJB2* gene. The frequency, therefore, was approximately 1 in 5.

**Conclusions:**

The data suggest that used blood routine and RDT can make a preliminary screening of sperm donors, and special gene testing should be performed for sperm donors according to the regional incidence of specific genetic diseases. Meanwhile, whole exome sequencing can be used as a supplementary application in sperm donor genetic testing, and aid a successful and healthy pregnancy. However, industry guidelines must be modified to incorporate its use.

## Introduction

Like all prospective parents, people who rely on donor sperm want to conceive healthy children. However, a retrospective study of birth defects and the medical risks of donor-conceived offspring found that approximately one-third of the birth defects in such offspring involved autosomal recessive (AR) disorders (1). In fact, some autosomal dominant (AD) disorders cannot always be detected at a donor’s eligibility assessment owing to reduced penetrance, variable expressivity, or temporal factors. Today, China has 27 sperm banks, the largest of which screens 4000 potential donors each year (2). Sperm donor screening is conducted strictly in accordance with standard guidelines published by the Chinese Ministry of Health in 2003 (3). One of the guidelines states that potential donors must undergo laboratory testing to exclude genetic diseases, and undergo karyotype analysis. However, numerous human sperm banks in China only perform three-generation family history evaluation to exclude genetic diseases with clinical symptoms. Therefore, many inherited risks cannot be detected before donor qualification even when a thorough genetic family history evaluation has been performed. Hence, not only is there a risk for autosomal recessive disease, but also risk for autosomal dominant variants with reduced penetrance, variable expressivity, or other complex modes of inheritance with the current eligibility guidelines in China for the donor selection process.

The American Society for Reproductive Medicine (ASRM) has recommended that sperm donors undergo appropriate genetic evaluation, e.g., genetic carrier screening (4). Fifteen of the 17 sperm banks in the United States reported that they would attempt to accommodate special requests from donor semen recipients to perform a particular genetic test on a specific anonymous sperm donor (5). In recent years, the cost of molecular genetic studies has been greatly reduced due to the emergence of next-generation sequencing (NGS) technologies, allowing the analysis of numerous genes and the concurrent evaluation of hundreds of variants (6). Some reproductive centers use NGS to ensure that donors and recipients do not have mutations in the same genes before assisted reproductive therapy (ART) is initiated, thereby avoiding high reproductive risk for transmitting a severe AR genetic condition to the offspring (7). Therefore, it is necessary to perform genetic testing of sperm before donor qualification.

In the present study, we performed genetic testing on donors to a human sperm bank in China. We used whole exome sequencing to analyze variants to determine if they are potentially pathogenic in the donor and recipient and in preassigned donor–recipient matches. We provide evidence that donors have a significant risk of passing on an inherited susceptibility for AR and undiagnosed AD disorders, and we recommend performing more genetic testing at human sperm banks.

## Patients and methods

### Study population and participants

This retrospective study reviewed the genetic test analyses and clinical outcomes of young adult men who were qualified sperm donors at the Hunan Province Human Sperm Bank of China from January 1, 2018, to May 1, 2021. Their clinical information was collected and analyzed, and included the clinical outcomes after semen use. All donors sign informed consent forms during their first visit to the sperm bank, consenting to the use of their semen samples or data by the sperm bank for scientific research purposes. A total of 3231 qualified sperm donors were included in this study: all donors underwent primary screening for thalassemia and glucose-6-phosphate dehydrogenase (G6PD) deficiency. Whereafter, 278 of donors underwent genetic testing for specific genes, and 43 donors underwent whole exome sequencing. The Central South University Ethics Committee approved this study (2021-KT48).

### Criteria for sperm donor screening in China

The guidelines for screening sperm donors in China are as follows (8): (a) donors must be between 22 and 45 years of age; (b) donors must be in good health, based on the results of a physical examination and a psychological evaluation by a qualified doctor, and have no familial history of a genetic disease; (c) fresh semen should have a liquefaction time of <60 min, sperm concentration ≥ 60 × 10^6^ mL^−1^, progressive sperm motility ≥ 60%, and normal morphology > 30%; (d) post-thaw semen should have motility of ≥40%, ≥12 × 10^6^ motile sperm per vial, and a frozen–thaw survival rate ≥ 60%; and (e) potential donors must undergo laboratory testing to exclude individuals at high risk for sexually transmitted infections and genetic diseases, including HIV-1 and HIV-2, hepatitis B and C, syphilis, gonorrhea, mycoplasma, chlamydia, cytomegalovirus, *Toxoplasma gondii*, rubella virus, and herpes simplex virus types 1 and 2, and undergo karyotype analysis. If the potential donor tests negative for all of the above tests and fulfills the Chinese Ministry of Health guidelines outlined above, the donation process is initiated and their semen samples are cryopreserved. The samples must be cryopreserved for a minimum 6-month quarantine period prior to rescreening for HIV.

### Criteria for management of donor sperm for external use in China

In China, sperm banks are required to maintain permanent records of the initial selection process and the subsequent tests and evaluations of each donor. The clinical outcomes (pregnant or not) for each treatment cycle (artificial insemination by donor [AID] and *in vitro* fertilization [IVF]) and fertility outcomes (abortion, healthy live birth, or birth defect) must also be recorded. Moreover, a sperm donor is permitted to impregnate a maximum of five women (2).

### Blood and DNA samples

The analyses were performed using blood and DNA samples collected for clinical purposes according to established protocols. 1.5ml blood samples were collected from qualified donors. DNA was extracted from the whole blood of the qualified sperm donors using commercially available DNA isolation kits (Gentra Systems, Minneapolis, MN, USA) following the manufacturer’s instructions.

### Premarital screening of thalassemia and G6PD

The blood sample for routine blood testing, hematology analyzers (HA) are used to measure the complete blood count which was screening sperm donors for thalassemia. G6PD deficiency Rapid Diagnostic Test (G6PD RDT, GuangZhou Jianlun biology Technology Co, Guangzhou, China) are used to screening sperm donors for G6PD deficiency. 2μl whole blood was placed into the sample window, immediately followed by two drops of buffer solution into the assay window according to the manufacturer’s instructions. After ten minutes at the room temperature, the RDT was visually read and classified as deficient or normal.

### Genetic testing

Genetic testing was performed *via* whole exome sequencing or for specific genes. Samples from all participants in this study were submitted to the Baylor Miraca Genetics Laboratory for clinical exome sequencing (CES, performed at the AmCare Genomics Laboratory, Guangzhou, China), which included coding exons for about 5000 clinically relevant disease-causing genes (9). The genomic DNA sequencing library was prepared by ultrasonic fragmentation, terminal repair, amplification, and purification, and the DNA sequence of the target region was enriched using custom-designed NimbleGen SeqCap probes (Roche NimbleGen, Madison, WI, USA). Then, the enriched DNA samples were sequenced on an Illumina HiSeq 2000 unit (Illumina, San Diego, CA, USA) with 150-bp single-end read length. All coding exons and 20 bp of the flanking intronic regions of the target genes were sequenced, with an average coverage depth of ~200×. The original raw data obtained from the Illumina platform were considered raw reads, and recorded in FASTQ files (fq). The data were filtered to generate clean reads by removing adapters and reads with base quality of <Q20. The sequencing reads were aligned to the human reference genome GRCh37/hg19. Specific gene testing only analyze regions of specific genes in the original raw data, while whole exome sequencing analyses all the regions in the original raw data. Single-nucleotide variants (SNVs) were observed with NextGENe software (version 2.4.1.2) (SoftGenetics, State College, PA, USA). Gene variants were annotated using population and literature databases, including the Genome Aggregation Database (gnomAD, https://gnomad.broadinstitute.org/), ClinVar (https://www.ncbi.nlm.nih.gov/clinvar/), and Online Mendelian Inheritance in Man (OMIM). Variants with minor allele frequency (MAF) > 1% in Asian populations were filtered out. Pathogenicity and evolutionary conservative analyses were performed using PolyPhen and VarCards. The pathogenicity of the gene variants was classified according to American College of Medical Genetics and Genomics (ACMG) guidelines (10). We eliminated low-quality sequencing data (the average coverage of the coding sequence was <3×) to analyze copy number variations (CNVs). The copy number changes of exons were analyzed with homogenization calculation; 1 indicated a normal level (×2), 0.5 indicated one missing copy (×1), and 0 indicated two missing copies (×0), that is, homozygous/haplotype deletion. The pathogenicity of the CNVs was evaluated by referring to databases and published literature such as the Database of Genomic Variants (DGV), DECIPHER, and OMIM. Sperm donors without clinical symptoms were considered positive if they carried a pathogenic or likely pathogenic variation in the gene of concern to the recipient. The web address of BioSample Database was https://www.ncbi.nlm.nih.gov/biosample/29624714-756.

### Interpretation of variants

All variants found were classified as pathogenic (pathogenic and/or likely pathogenic), common (polymorphisms), or of uncertain significance (10). Pathogenic variants were mutations classed as pathogenic or likely pathogenic in the ClinVar database and the Human Gene Mutation Database (HGMD, http://www.hgmd.cf.ac.uk), or described in the literature as associated with the disease. Variants of uncertain significance were variants not previously reported as pathogenic in the ClinVar database or HGMD and that presented frequencies in the population compatible with those of the disease of interest.

## Results

### Thalassemia and G6PD deficiency testing

That at the base of criteria for sperm donor screening in China, our sperm bank has increased the screening of thalassemia and G6PD deficiency for qualified sperm donors. The results as show in **Table 1**, 2.4% of qualified sperm donors might have thalassemia and 1.4% might have G6PD deficiency. Sperm donors with thalassemia and G6PD deficiency would be eliminated.

**Table 1:** Primary screening for thalassemia and G6PD with the whole blood of 3231 sperm donors.

Genetic disorder	Donors, n	Abnormal, n	Population proportion
Thalassemia (α+β)	3231	80	2.4%
Glucose-6-phosphate dehydrogenase deficiency	3231	56	1.4%

### Specific gene testing

**Table 2** summarizes the results for a part of genes that received requests for specific testing from donor semen recipients. Specific gene testing identified 7 of the 278 donors (2.5%) as carriers of at least one pathogenic or likely pathogenic variant in a gene, including 1.9% of 154 donors (3/154) as carrier variants in α-Like or β-Like globin genes, 17.6% of 17 donors (3/17) as carrier variants in *GJB2*, 12.5% of 8 donors (1/8) as carrier variants in *SMN1*. In addition, most of the requested genes were the α-like or β-like globin genes, followed by the genes related to G6PD deficiency, GJB2-related DFNB1 nonsyndromic hearing loss and deafness, and spinal muscular atrophy (*SMN1*). These were external-use donor semen recipients for more than 10 ART cycles. The remaining requested genes were *SLC26A4*, *HLAB27*, *PKHD1*, *ATPTB*, *TSCI*, *PAH*, and *ACSF3*.

**Table 2:** Genetic testing for specific disorder-related genes for 278 sperm donors in sperm donor applicants.

Gene	Genetic disorder	Inheritance	Donors, n	Abnormal, n	Abnormal proportion, n	Cycles, n	Pregnancies, n	Miscarriages, n	Deliveries, n
α-Like globin genes β-Like globin genes	Thalassemia	AR	154	2	1.3%	580	325	32	228
G6PD	Glucose-6-phosphate dehydrogenase deficiency	XD	23	1	4.3%	59	28	3	22
GJB2	GJB2-related DFNB1 nonsyndromic hearing loss and deafness	AR/AD	17	3	17.6%	23	7	1	5
SMN1	Spinal muscular atrophy	AR	8	1	12.5%	12	5	2	2
SLC26A4	Epicophosis	AR	4	0	0%	5	2	0	1
HLAB27	Ankylosing spondylitis	AD	3	0	0%	4	2	0	2
PKHD1	Polycystic kidney	AR	3	0	0%	3	0	0	0
ATP7B	Hepatolenticular degeneration	AR	2	0	0%	2	1	0	1
TSC1	Tuberous sclerosis complex (Bourneville-Pringle disease)	AD	2	0	0%	6	5	0	5
PAH	Phenylketonuria	AR	2	0	0%	6	5	1	4
ACSF3	Combined malonic and methylmalonic aciduria	AR	2	0	0%	5	4	2	2

Genes tested only once are not listed.

Autosomal Dominant (AD); Autosomal Recessive (AR); X-linked dominant (XD).

### Whole exome sequencing

**Figure 1:**
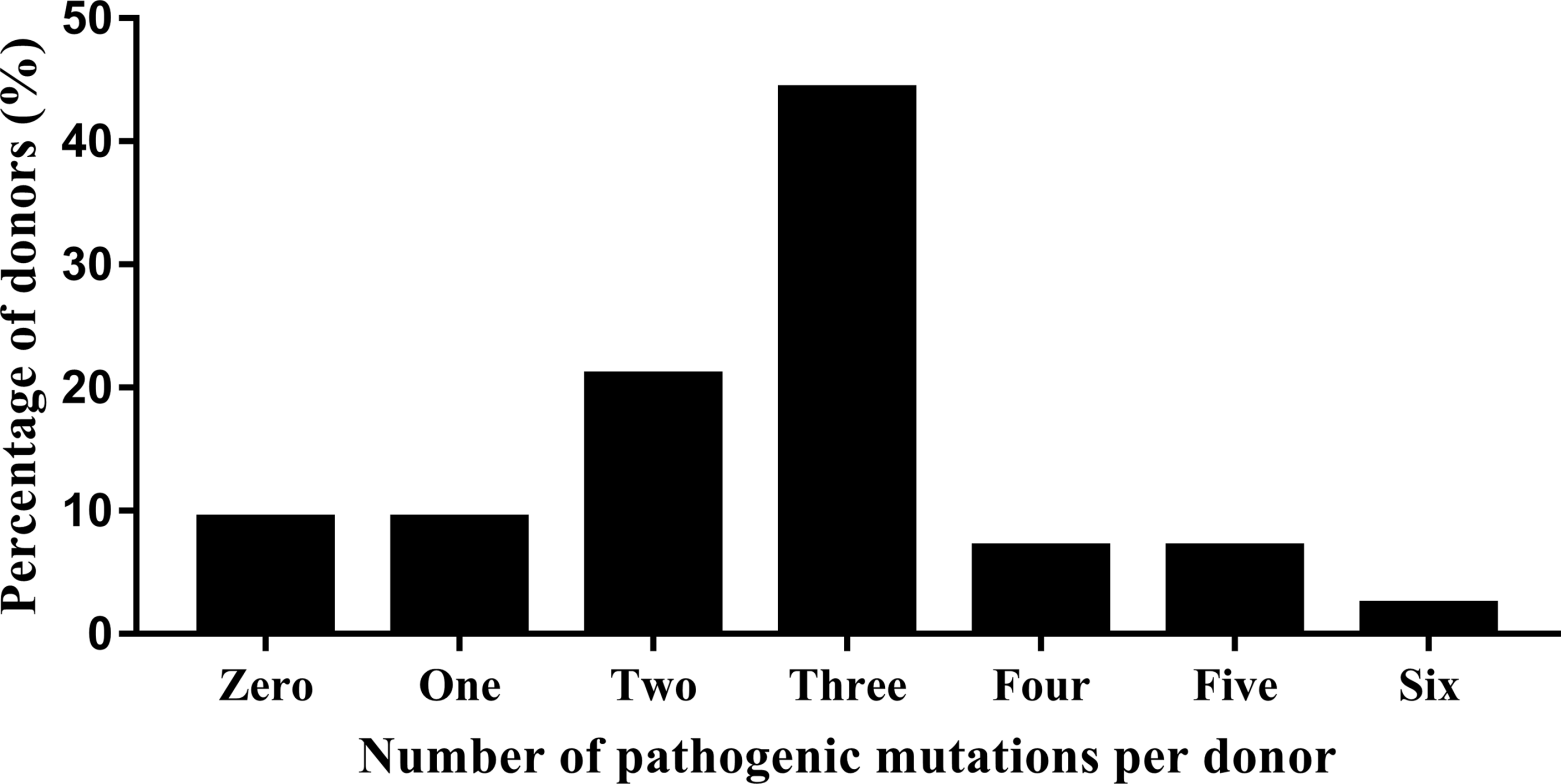
Whole exome sequencing detection of the number and percentage of pathogenic variants in 43 donors.

Whole exome sequencing in the clinical context of ART preconception screening identified 90.7% of the 43 donors (39/43) as carriers of at least one pathogenic or likely pathogenic variant in a gene responsible for a Mendelian genetic disease. Most of the donors were heterozygous for multiple variants (**Figure 1**); 92.3% (36/39) were carriers of ≥2 genetic variants. Only four donor was a carrier of only one variant (2.6%, 1/39). Most were heterozygous for only two or three variants (71.8%, 28/39) (**Figure 1**). The whole exome sequencing detected a total of six pathogenic variants (0% AD, 83.3% AR, 16.7% AR/AD, 0% Digenic Recessive (DR)), 105 likely pathogenic variants (3.8% AD, 73.3% AR, 20% AR/AD, 2.9% DR), and 184 variants of uncertain significance (21.2% AD, 61.4% AR, 16.8% AR/AD, 0.5% DR) in the 43 sperm donors (**Tables S1**, **3**). Among the 43 sperm donors carrying the 111 pathogenic/likely pathogenic variants, eight (18.6%) were carriers of pathogenic variants of the *GJB2* gene. The frequency, therefore, was approximately 1 in 5. Two of the 43 donors (4.65%) were carriers of pathogenic/likely pathogenic variants in the *SLC25A13*, *KDM5B*, *NEM8*, *DUOX2*, *CYP4V2*, *COQ8A*, *ROM1*, *DNAH1*, and *SERPINB7* genes; the frequency was approximately 1 in 20. Variants of pathogenic or likely pathogenic carriers in 43 sperm donors as shown in **Supplementary Table S2**, most of variants are common in Chinese.

**Table 3:** Whole-exome sequencing detection of identical carrying genes in 43 sperm donors.

Gene	Inheritance	Donors, n	Population proportion	Genetic disorder
GJB2	AR/AD	8	18.6%	GJB2-related DFNB1 nonsyndromic hearing loss and deafness
SLC25A13	AR	2	4.65%	Citrullinemia argininosuccinate synthase deficiency
KDM5B	AR	2	4.65%	AR intellectual disability
NME8	AR	2	4.65%	Late-onset Alzheimer disease
DUOX2	AR	2	4.65%	Thyroid dyshormonogenesis (dyshormonogenetic goiter)
CYP4V2	AR	2	4.65%	Bietti crystalline corneoretinal dystrophy
COQ8A	AR	2	4.65%	Coenzyme Q10 deficiency
ROM1	AR/AD	2	4.65%	Retinitis pigmentosa
DNAH1	AR	2	4.65%	Primary ciliary dyskinesia
SERPINB7	AR	2	4.65%	Palmoplantar keratoderma, Nagashima type

Autosomal Dominant (AD); Autosomal Recessive (AR).

### Clinical outcomes of carrier screening

Specific gene testing was performed on a total of 278 donors if requested by donor semen recipients. During the study period, 793 treatment ART cycles were completed, 433 clinical pregnancies were achieved, and 309 offspring were delivered. Meanwhile, whole exome sequencing was performed in a total of 43 donors, and we identified 45 cycles of preassigned donor–recipient matches with high reproductive risk for transmitting a severe AR genetic condition to the offspring and that required changing the initially selected donor for another without mutations in the same genes. We found two donor–recipient matches in which both were carriers for the same mutation, so the two recipients were matched to donors a second time. Twenty-five clinical pregnancies were achieved, and 18 offspring were delivered (**Supplementary Table S3**).

## Discussion

Here, the data from our human sperm bank showed that, other than three-generation family history evaluation, specific gene testing and whole exome sequencing can be used for the genetic testing of sperm donors.

In China, sperm donor eligibility at sperm banks is determined in part by infectious disease screening and family history risk assessment. Routine testing for genetic disease is becoming increasingly important as part of the donor selection process due to the rising number and expanding availability of genetic tests. Meanwhile, recipients’ demands for sperm donor genetic testing are also increasing, which has influenced donor screening and selection at human sperm banks. However, the sperm donor screening guidelines do not describe how genetic tests published by the Chinese Ministry of Health in 2003 (3), which are currently in effect (2), should be performed.

That at the base of criteria for sperm donor screening in China, sperm donors whose semen parameters up to standard would undergo further physical and laboratory examinations, including three-generation family history evaluation, karyotype analysis and so on. In addition, our sperm bank has increased the screening of thalassemia and G6PD for qualified sperm donors. Thalassemia is considered one of the most common genetic disorders resulting from globin chain synthesis impairment because of the mutation or deletion of globin gene, such as α- and β-thalassemia, with a high frequency in Southeast Asia (11). In recent years, large-scale surveys for thalassemia have been conducted in different parts of China, and its prevalence remains high (12, 13). A prevalence map based on a geographic information system (GIS) showed that the geographic distribution of thalassemia was highest in the south of China, including Guangdong, Guangxi, Guizhou, Yunnan and so on, and decreased from south to north (14). In the present study, we used blood routine testing to screening of thalassemia related gene carriers in qualified sperm donors, if blood routine results showed a decrease in mean corpuscular volume (MCV) and mean corpuscular hemoglobin (MCH), accompanied by an increase in red cell count (RBC), we would think that the sperm donors were suspected thalassemia related gene carriers, whom would be eliminated. Because of less globin production, the thalassemia RBC showed microcytic and hypochromic, MCV, MCH and RBC were valuable indicators for screening thalassemia related gene carriers (15–17). However, MCV, MCH and RBC cannot discriminate between α- and β-thalassemia, and cannot discover heterozygous thalassemia related gene carriers (18). Even so, 2.4% of sperm donors (80/3231) were suspected thalassemia related gene carriers, which was significantly lower than another study in Hunan province that analyzed by using Next-Generation Sequencing (19). G6PD deficiency is one of the most common X-linked enzymopathies caused by *G6PD* gene variants, and G6PD deficiency is a common genetic disease in China. The incidence of G6PD deficiency in China is also characterized by a high to low gradient distribution from the south to north regions (20). In the present study, we used RDT to diagnosis G6PD deficiency in qualified sperm donors, which can accurately identify hemizygous males and homozygous females. The data showed that 1.4% of sperm donors (56/3231) were G6PD deficiency whom would be eliminated. The overall prevalence of G6PD deficiency in China was 2.10% at the national level, and in Hunan province was 1.13% (21), which was similar to our results. Therefore, through the interpretation of blood routine and RDT results of sperm donors, it can make a preliminary screening of thalassemia and G6PD deficiency, and improve the laboratory examination of sperm donor genetic testing.

As early as 2010, all sperm banks in the United States have conducted some degree of genetic testing on their sperm donor applicants as part of the screening process. Furthermore, cystic fibrosis (CF) carrier screening, hemoglobin evaluation, and chromosome analysis are performed routinely for the majority of donor applicants (5). CF is one of the most common severe AR diseases in the white population, with an approximate incidence of 1 in 2500 live births (22). However, it is least common in Africans and Asians (23). Therefore, CF carrier screening is not available at human sperm banks in China. Our data show that sperm donor carrier screening for the thalassemia-related genes was the most requested by recipients. Meanwhile, recipient requests for sperm donor carrier screening for *G6PD* genes were only second to that for thalassemia. 1.9% of 154 donors (3/154) as carrier variants in α-Like or β-Like globin genes, although thalassemia and G6PD deficiency have been preliminary screened. This also shows while MCV, MCH and RBC were valuable indicators for screening thalassemia related gene carriers, genetic testing is the gold standard for diagnosing thalassemia related gene carriers. The incidence of genetic disease varies in different regions in China. Hence, genetic testing of sperm donors should be performed with targeted genetic disease-related genes of the different regions. In particular, donors of semen for external use in the Guangxi and Guangdong provinces in south China, which have a high incidence of thalassemia and G6PD deficiency, should be tested for the α-like, β-like globin genes and *G6PD* gene.

In the present study, carrier genetic testing was performed using NGS, which was first used in infertile couples wishing to conceive through ART in 2015. In the clinical dataset, 2161 samples (84%) tested positive, with an average carrier burden of 2.3 variants per sample. Five percent of the couples wishing to conceive through ART were carriers for the same mutation; genetic screening prevented the birth of 1.25% of genetically affected babies born after ART (24). Carrier genetic testing can be applied to couples wishing to conceive through ART, as well as ART with sperm or oocyte donors, to avoid serious monogenic genetic diseases (7). Comparison with the Exome Aggregation Consortium (ExAC) East Asian and European populations showed that the carrier frequency of disease-related genes identified at the Translational Medicine Center of the Children’s Hospital of Fudan University is much more similar to that of the East Asian population than the European population. The difference in carrier frequency among populations should not be ignored and makes it necessary to establish a Chinese-specific panel for genetic testing (25). In the present study, we performed whole exome sequencing carrier screening, which included more than 5000 genes associated with more than 7000 disorders in OMIM. We tested 43 sperm donors, with an average carrier pathogenic/likely pathogenic variant of 2.58 per sample. Forty-five cycles of preassigned donor–recipient matches were identified, 25 clinical pregnancies were achieved, and 18 offspring was delivered. Although the data are based on a limited number of participants, the results provide the carrier frequencies for many recessive disorders. Here, we found that, for many of the studied genes, the carrier frequencies for some of the most common recessive disorders, such as *GJB2*-related DFNB1 nonsyndromic hearing loss and deafness, citrullinemia argininosuccinate synthase deficiency, late-onset Alzheimer disease, and AR intellectual disability, are higher than that previously reported (26–29). This is not unexpected, as previous studies on carrier frequencies were based on known mutations, whereas here we explored the entire coding region of most genes. In addition, eight of 43 donors (18.6%) were carriers of pathogenic or likely pathogenic variants in *GJB2*; the frequency therefore was approximately 1 in 5. Meanwhile, in special gene testing, three of 17 donors (17.6%) also as carrier variants in *GJB2*. However, as different *GJB2* pathogenic variants are associated with various phenotypes of hearing loss. For instance, both c.109G>A homozygotes and compound heterozygous c.109G>A variants of GJB-2 indicate a significantly higher risk of developing hearing loss. Conversely, heterozygous c.109G>A variants alone do not increase the risk of hearing loss (30). In the present study, five of 8 variants were heterozygous c.109G>A variants, that do not increase the risk of hearing loss. Although the carrier frequencies of *GJB2* should not be over-interpreted, the frequency of *GJB2* variants should be a concern in sperm donors in China.

Whole exome sequencing and specific gene carrier screening are powerful tools, especially for certain high-risk ethnic populations. However, there is considerable debate within the sperm banking community as to which tests should be included in the donor application process, in part because genetic testing is expensive and adds considerably to the cost of qualifying as a sperm donor. Nonetheless, there is no guideline in China on the proper use of sperm samples after genetic testing, which also limits the application of genetic screening in sperm banks in China. Such as some sperm banks in China confused about whether to inform the donor of the results. We believe that it is unethical to withhold the genetic testing results from a donor, as they would be unaware of the risks of bearing progeny in the future. However, how to better inform the results to the sperm donors so as not to influence their lives is a question that needs to be answered. Meanwhile, many inherited risks cannot be detected before donor qualification even when a thorough genetic family history evaluation has been performed. Hence, genetic testing for sperm donors can improve the quality of donor screening effectively and reduce the genetic risks for the offspring by preassigned donor-recipient matches. Furthermore, genetic testing was required not only for sperm donors but also for recipients, aid a successful and healthy pregnancy. It is important that human sperm banks engage the services of genetics professionals so that their clients have access to counseling about their family’s medical histories and the value and limitations of genetic testing and its role in reducing the risk of birth defects in future offspring.

Our study has some limitations: we did not perform genetic testing of the donors’ offspring, as congenital abnormalities were not reported therein. Furthermore, our study is not based on the all general population wishing to conceive, as the study group consisted of sperm donors who were young men. Meanwhile, a lack of knowledge regarding the use of genetic testing outside China in the present study. Although 321 qualified sperm donors underwent genetic testing, a larger cohort would provide more information. More studies of this type are needed.

This is the first study to investigate genetic testing of donor specimens from a sperm bank in China. Our results are significant for sperm donors, recipients, and future offspring. The data clearly illustrate that the significant risks presented by donors include inherited susceptibility for AR and undiagnosed AD disorders, and suggest that used blood routine and RDT can make a preliminary screening of sperm donors, and special gene testing be performed for sperm donors according to the regional incidence of specific genetic diseases. Meanwhile, whole exome sequencing can be used as a supplementary application in sperm donor genetic testing, and aid a successful and healthy pregnancy. However, industry guidelines must be modified to incorporate its use.

## Data availability statement

The original contributions presented in the study are included in the article/**Supplementary Material**. Further inquiries can be directed to the corresponding authors.

## Ethics statement

The studies involving human participants were reviewed and approved by 2021-KT48. The patients/participants provided their written informed consent to participate in this study.

## Author contributions

Study conceptualization and design, patient recruitment, and data collection: CH, L-QF and W-BZ. Data analysis and manuscript drafting: CH and H-CN. Patient recruitment and data collection: H-LW and QL. Draft revision: W-JZ, Z-HH, X-FL, and Y-LT. All authors approved the final version of the manuscript.

## Funding

This work was supported by grants from the National Natural Science Foundation of China (grant number 82001634) and the China Postdoctoral Science Foundation (grand number 2019M661521). Scientic Research Foundation of the Health Committee of Hunan Province (20190862).

## Conflict of interest

The authors declare that the research was conducted in the absence of any commercial or financial relationships that could be construed as a potential conflict of interest.

## Publisher’s note

All claims expressed in this article are solely those of the authors and do not necessarily represent those of their affiliated organizations, or those of the publisher, the editors and the reviewers. Any product that may be evaluated in this article, or claim that may be made by its manufacturer, is not guaranteed or endorsed by the publisher.
