# Supplementary material for: Genetic testing of sperm donors at a human sperm bank in China
Source: Front Endocrinol (Lausanne). 2022 Sep 20;13:942447. doi: 10.3389/fendo.2022.942447 (PMC9530660; doi:10.3389/fendo.2022.942447)
Supplement: Supplementary file 1 [file DataSheet_1.doc]

**Supplementary TableS1 Whole-exome sequencing detection of variants in 43 sperm donors.**

| Variant | Pathogenic, n | Probably  Pathogenic, n | Uncertain significance, n |
| --- | --- | --- | --- |
| AD | 0 | 4 | 39 |
| AR | 5 | 77 | 113 |
| AR/AD | 1 | 21 | 31 |
| DR | 0 | 3 | 1 |
| Total | 6 | 105 | 184 |

Note: Autosomal Dominant (AD); Autosomal Recessive (AR); Digenic Recessive (DR)

**Supplementary Table S2 Variants of pathogenic or likely pathogenic carriers in 43 sperm donors**

| ID | Genes | Inheritance | Varients | Classification of varients |
| --- | --- | --- | --- | --- |
| 10939 | BBS12 | AR | c.2dupT (p.M1) | Likely Pathogenic |
|  | BCHE | AR | c.493delG (p.E165Kfs*5) | Likely Pathogenic |
|  | KIF1BP | AR | c.59C>A (p.S20*) | Likely Pathogenic |
| 10940 | CFHR1 | AR/AD | c.705C>G (p.Y235*) | Likely Pathogenic |
|  | GPR98 | AR/DR | c.4282_4288del（p.L1428Sfs*6) | Likely Pathogenic |
| 10936 | ROGDI | AR | c.805C>T (p.Q269*) | Likely Pathogenic |
| 10938 | GRHPR | AR | c.295C>T (p.R99*) | Likely Pathogenic |
|  | MMP1 | AR | c.988delG (p.A330Lfs*45) | Likely Pathogenic |
|  | SLC25A13 | AR | c.852_855del4 (p. M285Pfs*2) | Pathogenic |
| 10934 | AGXT | AR | c.1161C>A (p.C387*) | Likely Pathogenic |
| 10933 | CCNO | AR | c.258_262dup5 (p.Q88Rfs*8) | Likely Pathogenic |
|  | CYP4V2 | AR | c.802-8_810delinsGC | Likely Pathogenic |
|  | HYDIN | AR | c.2851C>T (p.R951*) | Likely Pathogenic |
| 10932 | GNAT1 | AR/AD | c.607G>T (p.E203*) | Likely Pathogenic |
|  | NR0B2 | AR/AD | c.293_301delinsAC (p.L98Hfs*6) | Likely Pathogenic |
|  | TMC1 | AR/AD | c.426G>A (p.W142*) | Likely Pathogenic |
| 10931 | BBS2 | AR | c.2107C>T (p.R703*) | Likely Pathogenic |
|  | CYP7B1 | AR | c.259+2T>C | Likely Pathogenic |
| 20654 | FMO3 | AR | c.172G>A (p.V58I) | Likely Pathogenic |
|  | ITGA6 | AR | c.3234dupA (p.Q1079Tfs*10) | Likely Pathogenic |
|  | PRPF8 | AD | c.1149delT (p.F383Lfs*6) | Likely Pathogenic |
| 20653 | SLC24A4 | AR/AD | c.1869+2G>A | Likely Pathogenic |
|  | TBCD | AR | c.205G>T (p.E69*) | Likely Pathogenic |
| 20652 | CUBN | AR | c.6471delT (p.N2157Kfs*59) | Likely Pathogenic |
|  | GJB2 | AR/AD | c.299_300delAT (p.H100Rfs*14) | Likely Pathogenic |
|  | KDM5B | AR | c.2474+1G>A | Likely Pathogenic |
|  | NME8 | AR | c.1059dupA (p.S354Ifs*13) | Likely Pathogenic |
|  | RIPPLY2 | AR | c.95+1G>A | Likely Pathogenic |
|  | USH2A | AR | c.15520-2A>T | Likely Pathogenic |
| 20651 | SERAC1 | AR | c.1798A>T (p.K600*) | Likely Pathogenic |
|  | VWA3B | AR | c.655G>T (p.E219*) | Likely Pathogenic |
|  | WDR72 | AR | c.536C>G (p.S179*) | Likely Pathogenic |
| 20650 | AIPL1 | AR | c.421C>T (p.Q141*) | Likely Pathogenic |
| 20649 | GORAB | AR | c.109_119dup (p.L41Nfs*2) | Likely Pathogenic |
|  | RNF168 | AR | c.671delA (p.D224Vfs*6) | Likely Pathogenic |
|  | RSPH9 | AR | c.722+2T>C | Likely Pathogenic |
| 20648 | KDM5B | AR | c.2474+1G>A | Likely Pathogenic |
|  | NT5E | AR | c.1438C>T (p.R480*) | Likely Pathogenic |
|  | SLCO1B1 | DR | c.1683-1G>T | Likely Pathogenic |
|  | SPTA1 | AD/AR | c.2671C>T (p.R891*) | Likely Pathogenic |
| 20647 | NEK1 | AR/DR | c.3700G>T (p.E1234*) | Likely Pathogenic |
| 22267 | CACNA2D4 | AR | c.846C>A (p.Y282*) | Likely Pathogenic |
|  | GJB2 | AD/AR | c.235delC (p.L79Cfs*3) | Pathogenic |
|  | RAD50 | AR | c.2979_2980delCA (p.H993Qfs*6) | Likely Pathogenic |
|  | PNPT1 | AR | 1-6 exon deleted | Likely Pathogenic |
|  | FLG | AD | c.11740C>T (p.Q3914*) | Likely Pathogenic |
| 22266 | CFAP43 | AR | c.3245_3246+1delAGG | Likely Pathogenic |
|  | SOHLH1 | AR/AD | c.346-1G>A | Likely Pathogenic |
| 22265 | REN | AD/AR | c.289delC (p.Q97Rfs*74) | Likely Pathogenic |
|  | UROC1 | AR | c.902+1G>A | Likely Pathogenic |
|  | FOXC2 | AD | c.1506+2G>T | Likely Pathogenic |
| 22264 | ACOX2 | AR | c.820-1G>A | Likely Pathogenic |
|  | ALPK3 | AR | c.192C>A (p.C64*) | Likely Pathogenic |
| 22263 | GJB2 | AR/AD | c.109G>A (p.V37I) | Likely Pathogenic |
|  | PJVK | AR | c.1059+2A>C | Likely Pathogenic |
|  | SLC10A2 | AR | c.291dupC (p.V98Rfs*66) | Likely Pathogenic |
| 22262 | ABCC2 | AR | c.2980delA (p.I994Lfs*29) | Likely Pathogenic |
|  | ATP2A1 | AR | c.1912C>T (p.R638*) | Likely Pathogenic |
|  | MPL | AD/AR | c.981-1G>C | Likely Pathogenic |
|  | NME8 | AR | c.793G>T (p.G265*) | Likely Pathogenic |
| 22261 | GJB2 | AD/AR | c.109G>A (p.V37I) | Likely Pathogenic |
|  | SLC34A2 | AR | c.1458+2T>A | Likely Pathogenic |
| 22260 | COQ8A | AR | c.1844dupG (p.S616Lfs*114) | Likely Pathogenic |
|  | FASTKD2 | AR | c.1302delT (p.G435Vfs*2) | Likely Pathogenic |
|  | SLC26A4 | AR | c.919-2A>G | Pathogenic |
| 30804 | CNTNAP1 | AR | c.2503C>T (p.R835*) | Likely Pathogenic |
|  | LSS | AR | c.1194+2T>C | Likely Pathogenic |
|  | SPINK5 | AR | c.2557C>T (p.R853*) | Likely Pathogenic |
| 30803 | DUOX2 | AR | c.602dupG (p.Q202Tfs*99) | Likely Pathogenic |
|  | GJB2 | AD/AR | c.109G>A (p.V37I) | Likely Pathogenic |
|  | SLC35A3 | AR | c.342+1G>T | Likely Pathogenic |
| 31815 | PUS3 | AR | c.55C>T (p.R19*) | Likely Pathogenic |
|  | ROM1 | AD/AR | c.724C>T (p.R242*) | Likely Pathogenic |
| 31814 | HARS2 | AR | c.928delC (p.L310*) | Likely Pathogenic |
|  | ILDR1 | AR | c.206delC (p.P69Lfs*33) | Likely Pathogenic |
|  | SLC22A12 | AR | c.151delG (p.A51Hfs*14) | Likely Pathogenic |
| 33208 | CCDC114 | AR | c.1715T>A (p.L572*) | Likely Pathogenic |
|  | COL17A1 | AR/AD | c.2165-2A>G | Likely Pathogenic |
|  | GJB2 | AD/AR | c.109G>A (p.V37I) | Likely Pathogenic |
|  | PNPLA1 | AR | c.1384+2T>C | Likely Pathogenic |
|  | PNPO | AR | c.448_451del (p.P150Rfs*27) | Likely Pathogenic |
| 34634 | DNAH1 | AR | c.7864C>T (p.R2622*) | Likely Pathogenic |
|  | RBM28 | AR | c.1744C>T (p.R582*) | Likely Pathogenic |
|  | ROM1 | AD/AR | c.339dupG (p.L114Afs*18) | Likely Pathogenic |
|  | SLC26A1 | AR | c.528C>A (p.Y176*) | Likely Pathogenic |
| 38441 | CYP24A1 | AR | c.845-2dupA | Likely Pathogenic |
|  | DUOX2 | AR | c.1239C>G (p.Y413*) | Likely Pathogenic |
|  | GJB2 | AD/AR | c.109G>A (p.V37I) | Likely Pathogenic |
| 39317 | CWC27 | AR | c.1002dupA (p.V335Sfs*13) | Likely Pathogenic |
|  | SFRP4 | AR | c.856-1G>T | Likely Pathogenic |
| 39316 | SERPINB7 | AR | c.796C>T (p.R266*) | Likely Pathogenic |
|  | SLC25A13 | AR | c.852_855delTATG (p.M285Pfs*2) | Pathogenic |
| 43888 | COQ8A | AR | c.1844dupG (p.S616Lfs*114) | Likely Pathogenic |
|  | DNAH9 | AR | c.3649delG (p.A1217Qfs*4) | Likely Pathogenic |
|  | GJB2 | AD/AR | c.109G>A (p.V37I) | Likely Pathogenic |
| 50173 | CLPB | AR | c.736+2T>C | Likely Pathogenic |
|  | DNAI2 | AR | c.1516C>T (p.R506*) | Likely Pathogenic |
|  | KIZ | AR | c.1144dupA (p.I382Nfs*3) | Likely Pathogenic |
| 58993 | CYP4V2 | AR | c.802-8_810delinsGC | Likely Pathogenic |
|  | DNAH1 | AR | c.3593del (p.D1198Afs*6) | Likely Pathogenic |
|  | SERPINB7 | AR | c.796C>T (p.R266*) | Likely Pathogenic |
| 58997 | MCCC2 | AR | c.592C>T (p.Q198*) | Likely Pathogenic |
|  | USP45 | AR | c.2105_2106delAT (p.H702Rfs*18) | Likely Pathogenic |
| 60190 | 16p13.3 |  | >4.4Kb dup | Pathogenic |
|  | CFTR | AR | 5T | Likely Pathogenic |
|  | GNE | AR/AD | c.18T>A(p.Y6*) | Likely Pathogenic |
|  | ITGB6 | AR | c.1496_1497delCA (p.T499Rfs*17) | Likely Pathogenic |
|  | PROKR2 | AD | c.533G>C (p.W178S) | Likely Pathogenic |
| 76966 | GALC | AR | c.1901T>C (p.L634S) | Pathogenic |
|  | HFE | AR | c.845G>A (p.C282Y) | Likely Pathogenic |
|  | NEFH | AR/AD | c.1357G>T (p.E453*) | Likely Pathogenic |

**Supplementary Table S3 Summary of clinical outcomes of sperm donor genetic testing.**

| Genetic test | Donors, n | Cycles, n | Pregnancies, n | Miscarriages, n | Deliveries, n |
| --- | --- | --- | --- | --- | --- |
| Specific gene testing | 278 | 793 | 433 | 45 | 309 |
| Whole-exome sequencing | 43 | 45 | 25 | 0 | 18 |
